# Our coronary revascularization strategy in a high-risk patient with total stenosis in bilateral femoral arteries and total laryngectomy

**DOI:** 10.1186/1749-8090-10-S1-A223

**Published:** 2015-12-16

**Authors:** Ufuk Yetkin, Yasemin Işik, Kazım Ergüneş, Köksal Dönmez, Ali Gürbüz

**Affiliations:** 1 Department of Cardiovascular Surgery, Katip Celebi University Izmir Ataturk Training and Research Hospital, Izmir, Turkey

## Background/Introduction

Due to technologic advancements in medicine, survival rates of malignancy patients are improving. This situation results with higher risk in interventions to cardiovascular system of elder patients.

## Aims/Objectives

Our case was 76 year-old male patient. Coronary angiography performed for chest pain complaint revealed serious coronary artery stenosis.

## Method

Cardiovascular surgery council decided surgery for patient and patient was hospitalized. Coronary angiography and distal terminal aortography which were performed in the same session, revealed50% stenosis in left common iliac artery and total occlusion of bilateral superficial femoral arteries. Transthoracic echocardiography examination revealed interventricular septum as 17 mm and pulmonary artery pressure as 30 mmHg. He had diabetes mellitus regulated with oral antidiabetics and chronic obstructive pulmonary disease, which was consulted to chest department and reported as medium-risk for surgery, in his medical history. 24 years before his admittance, he had total laryngectomy for laryngeal cancer. Patient was consulted to ear-nose-throat department preoperatively.

## Results

Patient underwent coronary artery bypass grafting surgery with these clinical findings. Intubation was performed directly from trachea with 7,0 mm spiraled tube. Proximal part of median sternotomy incision was left apart from laryngectomy for preventing contamination. Mean arterial pressure was stabilized over 70 mmHg during operation for preventing lower extremity ischemia and metabolic acidosis. Patient had successful coronary revascularization of three vessels (LAD, CxOM1, and RCApl) by using saphenous vein grafts. Patient recovered uneventfully. He was discharged with plan of hybrid intervention (PTA for left common iliac artery and bilateral femoro-popliteal bypass) 8-10 weeks later.

## Discussion/Conclusion

Bilateral serious peripheral artery disease dramatically increases the risk of surgical coronary revascularization. Risk is seriously elevated with total laryngectomy caused by cancer etiology. Multidisciplinary approach is essential in such cases. Morbidity and mortality may be reduced and safety of procedure may be increased with optimal pre and perioperative precautions.

## Consent

Written informed consent was obtained from the patient for publication of this Case report and any accompanying images. A copy of the written consent is available for review by the Editor-in-Chief of this journal.

